# Toward improving control performance of myoelectric arm prosthesis by adding wrist position feedback

**DOI:** 10.3389/fnhum.2022.905885

**Published:** 2022-07-19

**Authors:** Yue Zheng, Lan Tian, Xiangxin Li, Yingxiao Tan, Zijian Yang, Guanglin Li

**Affiliations:** ^1^CAS Key Laboratory of Human-Machine Intelligence-Synergy Systems, Shenzhen Institutes of Advanced Technology (SIAT), Chinese Academy of Sciences, Shenzhen, China; ^2^Shenzhen College of Advanced Technology, University of Chinese Academy of Sciences, Shenzhen Institute of Artificial Intelligence and Robotics for Society, Shenzhen, China; ^3^Guangdong-Hong Kong-Macau Joint Laboratory of Human-Machine Intelligence-Synergy Systems, Shenzhen, China

**Keywords:** myoelectric prosthesis, position feedback, myoelectric control, transradial amputee, vibrotactile

## Abstract

Wearing a myoelectric prosthesis is a basic way for limb amputees to restore their lost limb functions in the activities of daily living (ADLs). However, it is estimated that around 40% of amputees refuse the prosthesis. One of the primary reasons would be that the current prostheses lack appropriate sensory feedback. Currently, the amputees only depend on their visual feedback (Vis-FB) when using their arm prostheses. It would be difficult for them to accurately control the wrist position, which is vital for flexible manipulation in ADLs. This manuscript designed a myoelectric arm prosthesis with wrist position feedback (WP-FB). To study the effect level of position feedback on prosthetic control, two tests were performed. The vibrotactile perception range test aims to analyze the perception sensitivity of the vibration in humans and obtain the optimal perception range utilized in the sensory feedback test. The sensory feedback test analyzes the effectiveness of the position feedback by comparing three feedback methods of Vis-FB, WP-FB, and a combination of Vis-FB and WP-FB (VP-FB). These tests were conducted by asking six able-bodied subjects to perform 20 movement combinations of five target positions. The WP-FB was transiently activated with five vibrating motors embedded in an armband to stimulate the arm stump when the prosthetic wrist rotates to the target positions. Our experimental results showed that when WP-FB was added to the prosthetic control, the absolute angular error (AAE) of the prosthetic wrist declined from 4.50° to 1.08° while the success rate 3 (SR3) increased from 0.34 to 0.84, respectively. This study demonstrates the importance of WP-FB to the effective control of the arm prosthesis.

## Introduction

Wearing a myoelectric prosthesis is a critical approach to recovering the lost limb functions of amputees in the activities of daily living (ADLs) (Kashef et al., 2020). The original intention of the prosthesis design is to restore the ADLs for amputees and enable them to return to social life (Zheng et al., 2018). However, about 40% of existing amputees refuse to use prostheses (Biddiss and Chau, 2007b; Ostlie et al., 2012). One of the main reasons is that some amputees believe that sensory feedback in the stump is more important than the prosthesis function (Biddiss and Chau, 2007a). In addition, visual feedback (Vis-FB) is still the primary feedback method for the existing prostheses. Over-reliance on visual cues resulting from a lack of appropriate sensory feedback makes the effective closed-loop control of prostheses difficult, affecting the control accuracy of prostheses and proprioception for amputees (Lewis et al., 2012). The closed-loop control of myoelectric prosthesis usually includes efferent and afferent signaling pathways. The efferent signaling pathway acquires myoelectric signals (EMG) from muscle residues, classified and decoded to control the prosthesis (Li et al., 2021). The afferent signaling pathway provides proprioception, the sense of limb information such as force, position, and temperature to the amputee, which is predominantly absent from current commercial prosthetic systems (D’Anna et al., 2019).

To recover the lost proprioception resulting from amputation for amputees, many researchers have tried to offer multiple sensory information of force, position, and temperature for amputees with various substitution methods. These methods can be divided into invasive and non-invasive sensory feedback methods (Svensson et al., 2017). Invasive sensory feedback, such as target muscular nerve reconstruction (Kim and Colgate, 2012) and neural electrode interface technology (Oddo et al., 2016), usually requires surgical intervention. Invasive to the human body limits its promotion (Stephens-Fripp et al., 2018). Non-invasive sensory feedback conveys sensory information to the user without surgical intervention. Although the sensory information provided by non-invasive methods is limited, it is less harmful and relatively simple. Furthermore, there are more choices of sensory substitution methods for non-invasive sensory feedback, which is enough to support a certain degree of functions. Therefore, there are many studies on non-invasive sensory feedback (Stephens-Fripp et al., 2018). Non-invasive sensory feedback commonly includes vibrotactile feedback (Chaubey et al., 2014), temperature feedback (Cho et al., 2007), mechanical tactile feedback (Godfrey et al., 2016), electrotactile feedback (Hartmann et al., 2014; Franceschi et al., 2015), acoustic feedback (Wilson and Dirven, 2017), and augmented reality feedback (Markovic et al., 2017).

Vibrotactile feedback is commonly used due to its advantages of small size and lightweight (Gu et al., 2021). Studies have shown that the two most essential sensory information proposed by amputees are force and position (Hermens et al., 2011). The Vib-FB has been used to provide grasp perception in many existing studies (Li et al., 2016; Yamada et al., 2016; Markovic et al., 2018; Niwa et al., 2021; Johnson et al., 2022). Vib-FB has been used in the virtual elbow angular position control (Wheeler et al., 2010; Hasson and Manczurowsky, 2015) and virtual wrist angular position control (Bark et al., 2010; Bensmaia et al., 2015). However, few studies analyze the wrist angular position feedback control (WP-FB) with the practical prosthetic wrist, which will be explored in this study.

The wrist position is essential for flexible manipulation in ADLs. For example, writing, reading, and typing on a computer require the wrist to keep in an almost fixed position, while opening a door with keys or pouring water from one glass to another asks for the continuous movement of the wrist (Vega-Gonzalez et al., 2007). Therefore, a prosthetic wrist with accurate control performance would help amputees regain some of their capacity in ADLs.

A myoelectric arm prosthesis was designed in this manuscript, which includes a self-designed arm prosthesis, a battery-powered EMG acquisition and control system, and a vibrotactile armband. To study the effect level of position feedback on prosthetic control, two tests were performed based on the designed myoelectric arm prosthesis. The vibrotactile perception range test aims to analyze the perception sensitivity of the vibration in humans. The sensory feedback test analyzes the effectiveness of the position feedback by comparing three feedback methods of Vis-FB, WP-FB, and a combination of Vis-FB and WP-FB (simplified as VP-FB hereafter). The completion time (CT), the absolute angular error (AAE), and the success rate (SR) were used to estimate the control performance of the arm prosthesis.

## Materials and methods

### Subjects

Six able-bodied subjects (four males and two females, 28 ± 4 years) were recruited for the experiments. The experimental protocol was approved by the Institutional Review Board of Shenzhen Institute of Advanced Technology, Chinese Academy of Sciences. All subjects have given written informed consent and provided permission for the publication of photographs for scientific and educational purposes.

### Experimental procedure

**FIGURE 1:**
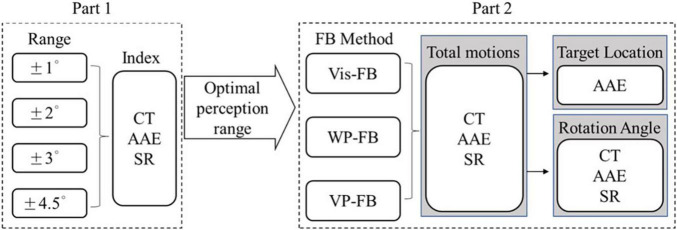
The experiment procedure.

A myoelectric arm prosthesis with WP-FB was designed and tested in the practical experiment. The experiment consists of two parts: part 1 is the vibrotactile perception range test, and part 2 is the sensory feedback test. The vibrotactile perception range test aims to analyze the perception sensitivity of the vibration in humans and obtain the optimal perception range utilized in the sensory feedback test. The perception range means the feedback range of vibration, which will incredibly influence the control performance of the prosthesis. Four ranges of ±1°, ±2°, ±3°, and ±4.5° were tested and compared to achieve the optimal perception range used in the sensory feedback test. To analyze the effectiveness of the position feedback, three feedback methods of Vis-FB, WP-FB, and VP-FB were performed with the optimal perception range in the sensory feedback test. These tests were conducted by asking six able-bodied subjects to perform 20 movement combinations of five target positions. The experimental results of the sensory feedback test were analyzed from three perspectives. The first perspective analyzes the 20 movements as a whole, while the other two perspectives classify 20 movements according to different target positions and rotation angles before analysis, respectively. The CT, AAE, and SR were utilized to compare the control performance of the arm prosthesis under different perception ranges and feedback methods. The experiment procedure is displayed in Figure 1.

#### Target position

**FIGURE 2:**
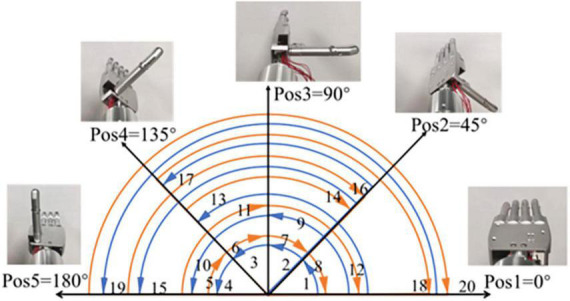
The five target positions and 20 movements of the experiment.

The experiment selected five commonly used angular positions of the wrist as target positions, named position 1 to position 5 (simplified as Pos1 to Pos5). Twenty movement combinations of the five target positions need to be completed in the vibrotactile perception range and sensory feedback tests. The angle interval between the adjacent positions is 45°. The starting and ending positions, the specific sequence, and the relative angle of each position of 20 movements are displayed in Figure 2. Each target position needs to be reached four times. The prosthetic hand utilized in the experiment is a left hand. Therefore, Pos1 is defined as the original position (0°), which represents the back of the prosthetic hand upward. Pos5 is defined as the final position (180°), which means the prosthetic hand palm up. The five target positions were utilized for practical considerations. These five positions are commonly used to help complete ADLs. For example, 90° is used in object grasping, 0° is useful in pouring water, and 180° is useful in delivering objects. Another two middle angular positions of 45° and 135° were also added to better discriminate wrist positions.

#### Vibrotactile feedback mode

**FIGURE 3:**
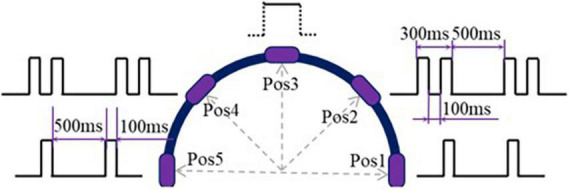
Vibrotactile feedback mode.

The vibrotactile mode used in WP-FB was designed to make subjects clearly distinguish five target positions of the prosthetic wrist. After considering the vibrating position and method, the vibrotactile modes used to encode the five positions are shown in Figure 3. In Pos1 and Pos5, the vibrotactile mode is the cycle of vibrating 100 ms and pausing 500 ms. In Pos2 and Pos4, the vibrotactile mode is the cycle of vibrating 100 ms, pausing 100 ms, vibrating 100 ms, and then pausing 500 ms. In Pos3, the vibrotactile armband keeps vibrating once the prosthetic wrist is within the perception range of the target positions. Each vibrating motor corresponds to one position. Once the prosthetic wrist moves into the preset perception range of the target positions, the vibration is transiently activated with the vibrating motor embedded in a vibrotactile armband to stimulate the arm stump. When the prosthetic wrist moves out of the preset range of the target positions, the vibration stops.

#### Vibrotactile perception range test

The perception sensitivity of the vibration in humans was studied by comparing the control performance of the arm prosthesis under four different perception ranges. The vibrotactile perception range test selected four ranges of ±1°, ±2°, ±3°, and ±4.5°. The subjects indicated difficulty perceiving vibration if the perception range was less than 1°. In addition, a perception range greater than 4.5° will lead to an error larger than 10% of the angle interval, which should be avoided in the experiment. Therefore, four ranges between 1° and 4.5° were selected and tested in the vibrotactile perception range test.

**FIGURE 4:**
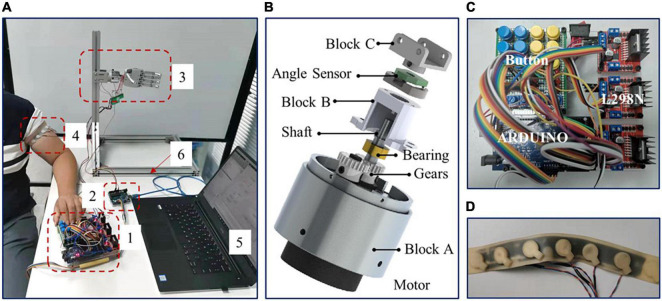
**(A)** The devices of vibrotactile perception range test, **(B)** the prosthetic wrist, **(C)** button control module, and **(D)** vibrotactile armband.

The devices of the vibrotactile perception range test are shown in Figure 4A, including the following six parts: (1) Button control module; (2) Angle acquisition module; (3) Self-developed arm prosthesis; (4) Vibrotactile armband; (5) Upper computer; (6) Support bracket.

The self-developed arm prosthesis utilized in this test includes a 6-DOF prosthetic hand (350 g) and a 1-DOF rotatory wrist (150 g), with a total weight of about 500 g. The prosthetic wrist is shown in Figure 4B. The prosthetic wrist adopts one eccentric shaft reduction motor, which can rotate 360°. The motor speed of the prosthetic wrist is about five rotations per minute, and the wrist diameter is 40 mm. The rotation center of the motor is adjusted to the center of the prosthetic wrist through gears with a reduction ratio of 1. The position of the prosthetic wrist is obtained by angle sensor V01A103AEA01R00 (Murata), and Block C is used to connect the prosthetic wrist and hand with screws.

The button control module is shown in Figure 4C. The module adopted Arduino to collect wrist angles and converted the angle into a corresponding vibrotactile mode to control the vibrotactile armband. Subjects operated the rotation of the prosthetic wrist by pressing two control buttons. The button signal was then collected by Arduino and then transferred to chip L298N to drive the prosthetic wrist. Meanwhile, Matlab@2010b software in the upper computer acquired and saved the feedback data of wrist angular positions from the angle acquisition module by serial port.

The vibrotactile armband is displayed in Figure 4D. The vibrotactile armband is made of 3D printed soft rubber, mounted with five brush flat vibrating motors. The motors of pos2 and pos4, and pos1 and pos5, were symmetrically placed on the ventral and rear aspects of the left upper arm, about 8 cm from the elbow joint, respectively. The motor of pos3 was aligned to the intersection of the coronal plane and upper arm. The frequency, diameter, and thickness of the motor are 30 Hz, 10 mm, and 3.4 mm, respectively.

The experimental process of the vibrotactile perception range test is set as follows: The arm prosthesis was first mounted on the support bracket and adjusted to the initial position before the test. Then, a vibrotactile armband was tied to the left upper arm of the subject. In addition, subjects wore eye masks to shield their vision. Three minutes were left to familiarize subjects with vibrotactile modes. Then, the subjects were asked to operate the prosthetic wrist to complete 20 movement combinations of five target positions through the button control module for each perception range. The experimenter told the subject the target positions of each movement one by one. The subject operated the prosthetic wrist once he knew the target position and stopped once he regarded the target position was reached and simultaneously told the experimenter that he had completed the movement. During the test, the angular position of the prosthetic wrist was obtained and displayed on the upper computer, which could be read by the experimenter in real-time. Suppose the angular position of the prosthetic wrist excessed the error threshold, in that case, the initial position of the next movement will be adjusted to the target position by the experimenter with a DC power supply. Each vibrotactile perception range test was repeated twice. The sequence of the total eight perception range tests was randomized, and the subjects did not know the current perception range.

#### Sensory feedback test

**FIGURE 5:**
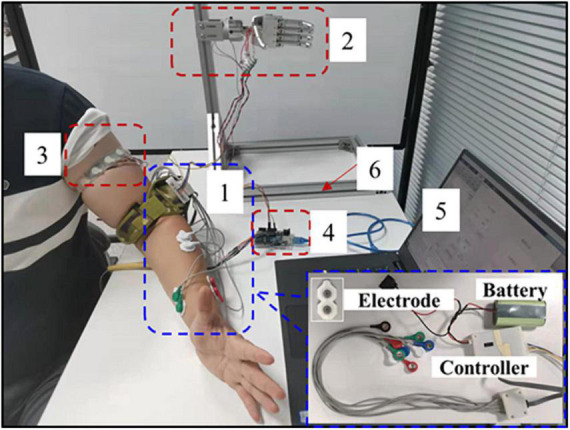
The devices of sensory feedback test.

The effectiveness of the position feedback on prosthetic control was tested in the sensory feedback test. The Vis-FB, WP-FB, and VP-FB were utilized as feedback methods to control the arm prosthesis. The devices of sensory feedback test are shown in Figure 5, including the following parts: (1) Self-developed EMG acquisition and control system; (2) Self-developed arm prosthesis; (3) Vibrotactile armband; (4) Angle acquisition module; (5) Upper computer; and (6) Support bracket.

A self-developed EMG acquisition and control system powered by a battery was designed to collect and classify the EMG signals and control the arm prosthesis in real-time. The experimental process of the sensory feedback test is set as follows: The arm prosthesis was first installed on the support bracket and adjusted to the initial position. Four EMG electrodes were affixed to the subject’s left forearm, over brachioradialis, the finger and wrist flexor, and extensor muscles. The exact positions of the electrodes were decided by palpating the contracted muscles. The reference electrode was placed on the olecranon of the same arm. The EMG signals of two motion classes of hand open/close motions were used to control the prosthetic wrist. Linear discriminant analysis was utilized to classify the EMG signals. The EMG signal was used as a binary signal. If the collected EMG signals were classified as the motion class of hand open, the prosthetic wrist would be operated to rotate externally, whereas if the collected EMG signals were classified as the motion class of hand close, the prosthetic wrist would be operated to rotate internally.

After the devices were set up, a brief training session was performed to ensure that the myoelectric control performance was satisfactory. The subjects were asked to control the prosthetic wrist to rotate externally and internally several times. As four-channel EMG signals were used to classify two classes of motions, the classification success rate was about 98%. Besides, the control system shows no visible delay. Because the subject could flexibly and correctly operate the prosthetic wrist with the hand open/close at a comfortable muscle contraction level, the control was deemed good, and the following session could proceed.

The vibrotactile armband was tied to the left upper arm of the subject. Three minutes were left to familiarize subjects with vibrotactile modes. Subjects were asked to operate the prosthetic wrist to complete 20 movement combinations of five target positions for each feedback method. The control of the movements is similar to that of the vibrotactile perception range test. Each test was repeated twice. The sequence of the total six feedback tests was randomized. In the tests with Vis-FB and VP-FB, subjects used EMG signals to control the arm prosthesis without shielding their vision, while in the test with WP-FB, subjects were asked to wear an eye mask to shield their vision. The upper computer recorded the results.

### Data analysis

#### Vibrotactile perception range test

In the vibrotactile perception range test, the purpose of data analysis was to study the influence of different perception ranges on the control performance of the arm prosthesis under WP-FB by comparing the indexes of CT, AAE, and SR. The CT means the completion time of each movement. The AAE means the absolute angular error between the actual and target positions. The SR was influenced by the CT and AAE. During the test, when the AAE exceeds a specified angle threshold, the running movement is considered to be failed. Similarly, the movement is also considered a failure when CT exceeds a given time threshold. The angle and time thresholds were calculated by the interquartile range (IQR). The AAE of all movements in the first perception range was pooled together, and the value of Q75 + 1.5*IQR was taken as angle threshold. The CT of all movements in four kinds of perception ranges was pooled together, and the value of Q75 + 1.5 × IQR was taken as the time threshold. Furthermore, the number of movements to be completed in each test is denoted as the number of total movements (NTM). The number of movements that exceed the time threshold and angle threshold during each test was recorded as NMT (number of movements exceeding time threshold) and NMA (number of movements exceeding angle threshold), respectively. If a movement exceeded both the time and the angle thresholds, the movement would be recorded as a failure only once. The SR was subdivided into SR1, SR2, and SR3, which represent the success rates when movements exceed the time threshold, the angle threshold, and both the time and the angle thresholds in the test, respectively. The SR1, SR2, and SR3 can be calculated by Eqs (1–3). The SR equal to 1 means no failure movement exists in the experiment. The outcome measures of AAE, CT, and SR were computed for each subject in each of the four perception ranges and the results were then pooled across all subjects with respect to perception ranges.


(1) SR1=(NTM-NMT)/NTM

(2) SR2=(NTM-NMA)/NTM

(3) SR3=(NTM-NMT-NMA)⁢/⁢NTM

#### Sensory feedback test

The sensory feedback test aims to study the effectiveness of the position feedback by comparing the control performance of the arm prosthesis under different feedback methods. The CT, AAE, and SR were used to evaluate the control performance of the prosthesis. The experimental results were analyzed from three perspectives. From the first perspective, the difference among the three feedback methods on prosthetic control was analyzed by comparing the CT, AAE, and SR of 20 movements. The outcome measures of AAE, CT, and SR were computed for each subject in each of the feedback methods and the results were then pooled across all subjects with respect to feedback methods. From the second perspective, to analyze whether the influence of the feedback method on the prosthetic control was related to the position, the 20 movements were classified according to five target positions. From the third perspective, to analyze whether the influence of the feedback method on the prosthetic control was related to the rotation angle, the 20 movements were divided into four groups according to four different rotation angles. These four groups were named according to the rotation angle values, which were R45° (rotation 45°), R90° (rotation 90°), R135° (rotation 135°), and R180° (rotation 180°). When the 20 movements were grouped according to rotation angles, the data amount of each group was not equal. To reduce the influence produced by the difference of data amount on statistical analysis, the data amounts of different rotation angles were changed to be equalized by selecting part of the data from the group of R45°, R90°, and R135° randomly. Then, the data amounts of R45°, R90°, and R135° were equal to that of the R180°. Finally, the data amount of each group was 2 (times test) × 6 (subjects) × 2 (times R180°) = 24. The outcome measures of AAE, CT, and SR were computed for each subject in each target position and rotation angle and the results were then pooled across all subjects with respect to their feedback methods (Vis-FB, WP-FB, and VP-FB) and grouping factors (target position and rotation angle).

Since the data from the vibrotactile perception range test and sensory feedback test both failed to pass the normal distribution test (Lilliefors test), the Friedman test was utilized to reveal a statistically significant effect. The Wilcoxon sign-rank test was used as a post-hoc pairwise test. The results are reported as median [inter-quartile range (IQR)]. Statistical significance was set as *p* < 0.05 in this work.

## Results

### Vibrotactile perception range test

**FIGURE 6:**
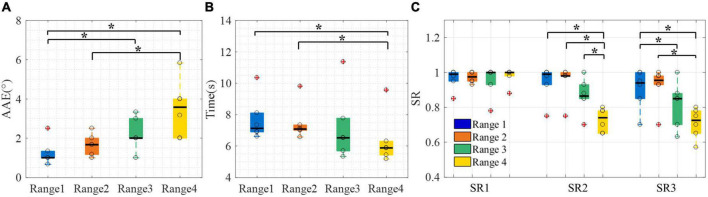
The control performance of the arm prosthesis with different perception ranges. **(A)** AAE, **(B)** CT, **(C)** SR. Boxplots depict the median (*line*), interquartile range (*box*), maximal/minimal values (*whiskers*), and outliers (*crosses*). The hollow circle denotes the median AAE, CT, and SR of each subject in panels **(A–C)**, respectively. A star denotes the statistically significant differences (*p < 0.05). AAE, absolute angular error; CT, completion time; SR1 to SR3, success rate when considering time threshold, angle threshold, and combination of the time and angle thresholds, respectively.

Four kinds of perception ranges of ±1°, ±2°, ±3°, and ±4.5° were represented by range 1 to range 4, respectively. The results of AAE, CT, and SR with different perception ranges are shown in Figure 6. From Figures 6A,B, there was a significant difference across four perception ranges for both AAE (DoF = 3, χ^2^ = 16.53, *p* < 0.001) and CT (DoF = 3, χ^2^ = 9.81, *p* < 0.05). The AAE increased with the increase of the perception range, while the CT decreased with the increase of the perception range. The AAE of four perception ranges were 1.00 (0.33), 1.67 (0.83), 2.00 (1.00), and 3.58 (2.00) degrees, respectively. The CT of four perception ranges were 7.12 (1.22), 7.06 (0.34), 6.52 (2.08), and 5.88 (0.88) s, respectively. The post-hoc analysis determined that the AAE of range 1 was significantly different from that of range 3 and range 4. Besides, the AAE of range 2 was significantly different from the AAE of range 4. The CT of range 4 showed a significant difference from that of range 1 and range 2. It can be seen from Figure 6C that SR1 showed no significant difference among all perception ranges, while there was a significant difference across four perception ranges for both SR2 (DoF = 3, χ^2^ = 13.58, *p* < 0.01) and SR3 (DoF = 3, χ^2^ = 10.44, *p* < 0.05). The SR2 of range 4 was significantly different from that of the other three ranges. The SR3 of range 4 was significantly different from that of range 1 and range 2. Besides, the SR3 of range 1 was significantly different from that of range 3.

To sum up, the vibrotactile perception range test showed that the AAE has no significant difference between ranges 1 and 2, and the CT has no significant difference among ranges 1 to 3. Besides, the SR3 of range 1 [0.94 (0.15)] was not significantly different from that of range 2 [0.96 (0.05)]. Therefore, ranges 1 and 2 were both appropriate for the following test. Finally, range 2 was selected to be utilized in the sensory feedback test because it had a slight advantage in SR3.

### Sensory feedback test

**FIGURE 7:**
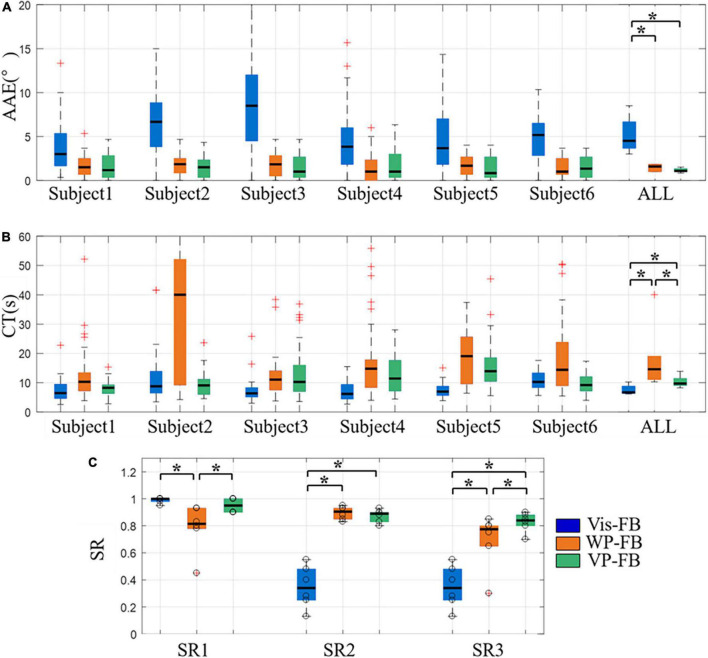
The control performance of the arm prosthesis under different feedback methods, **(A)** AAE, **(B)** CT, **(C)** SR. Boxplots depict the median (*line*), interquartile range (*box*), maximal/minimal values (*whiskers*), and outliers (*crosses*). The hollow circle denotes the results of each subject in panel **(C)**. A star denotes the statistically significant differences (*p < 0.05). AAE, absolute angular error; CT, completion time; SR1 to SR3, success rate when considering time threshold, angle threshold, and combination of the time and angle thresholds, respectively. ALL represents the result when the data of all subjects were pooled together to calculate the control performance for each feedback method.

Under Vis-FB, WP-FB, and VP-FB, the control performance of the arm prosthesis was compared using indexes of AAE, CT, and SR. The experimental results were analyzed from three perspectives. The results of the first perspective are displayed in Figure 7.

Figures 7A,B show the AAE and CT under three feedback methods, respectively. The overall performance of AAE under Vis-FB, WP-FB, and VP-FB was 4.50 (3.00), 1.58 (0.83), and 1.08 (0.33) degrees, respectively. The overall performance of CT under Vis-FB, WP-FB, and VP-FB was 6.69 (2.42), 14.57 (8.01), and 9.71 (2.37) s, respectively. There was a significant difference across three feedback methods for both AAE (DoF = 2, χ^2^ = 10.17, *p* < 0.01) and CT (DoF = 2, χ^2^ = 10.33, *p* < 0.01). The post-hoc analysis determined that when considering the overall performance of all subjects, the AAE of Vis-FB was about three times larger than that of WP-FB and VP-FB and this difference was statistically significant. The difference in AAE among the three feedback methods was similar across all subjects. When considering the overall performance of all subjects, the CT of WP-FB, Vis-FB, and VP-FB were significantly different pairwise. The CT of WP-FB showed a significant difference from that of Vis-FB and VP-FB in three subjects, while the CT of Vis-FB was significantly different from that of WP-FB and VP-FB in another three subjects.

Figure 7C shows the SR1 to SR3 under three feedback methods. There was a significant difference across three feedback methods for SR1 (DoF = 2, χ^2^ = 9.64, *p* < 0.05), SR2 (DoF = 2, χ^2^ = 9.00, *p* < 0.05), and SR3 (DoF = 2, χ^2^ = 9.33, *p* < 0.01). The post-hoc analysis determined that the SR1 [0.82 (0.15)] of WP-FB was significantly smaller than that of Vis-FB [1.00 (0.02)] and VP-FB [0.95 (0.10)], while the SR2 of Vis-FB [0.34 (0.23)] was significantly smaller than that of WP-FB [0.91 (0.08)] and VP-FB [0.89 (0.07)]. The SR3 of three feedback methods were 0.34 (0.23), 0.78 (0.15), and 0.84 (0.08), respectively. The SR3 of the three feedback methods were significantly different pairwise.

**FIGURE 8:**
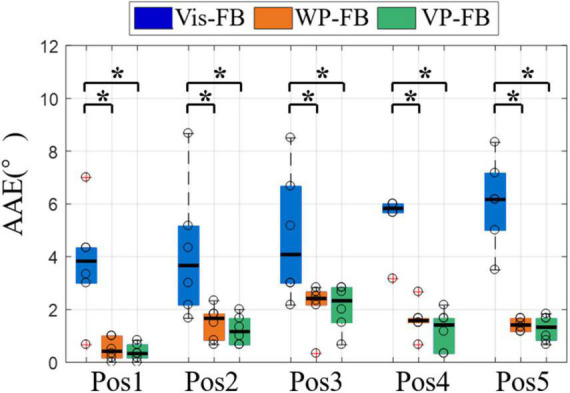
The AAE of five target positions under three feedback methods. Boxplots depict the median (*line*), interquartile range (*box*), maximal/minimal values (*whiskers*), and outliers (*crosses*). The hollow circle denotes the median AAE of each subject. A star indicates the statistically significant differences (*p < 0.05). AAE, absolute angular error.

The results from the second perspective are displayed in Figure 8, which shows the AAE of five target positions under three feedback methods. There were significant differences across three feedback methods for all target positions (DoF = 2, χ^2^ = 7.91, 7.00, 7.64, 9.33, and 9.00 for five target positions, respectively, *p* < 0.05). The AAE of Vis-FB was significantly higher than that of WP-FB and VP-FB at all target positions, while there was not a significant difference between WP-FB and VP-FB. This is in accordance with the performance of AAE when considering the total movements. Besides, there is no significant difference among different target positions in the same feedback method.

**FIGURE 9:**
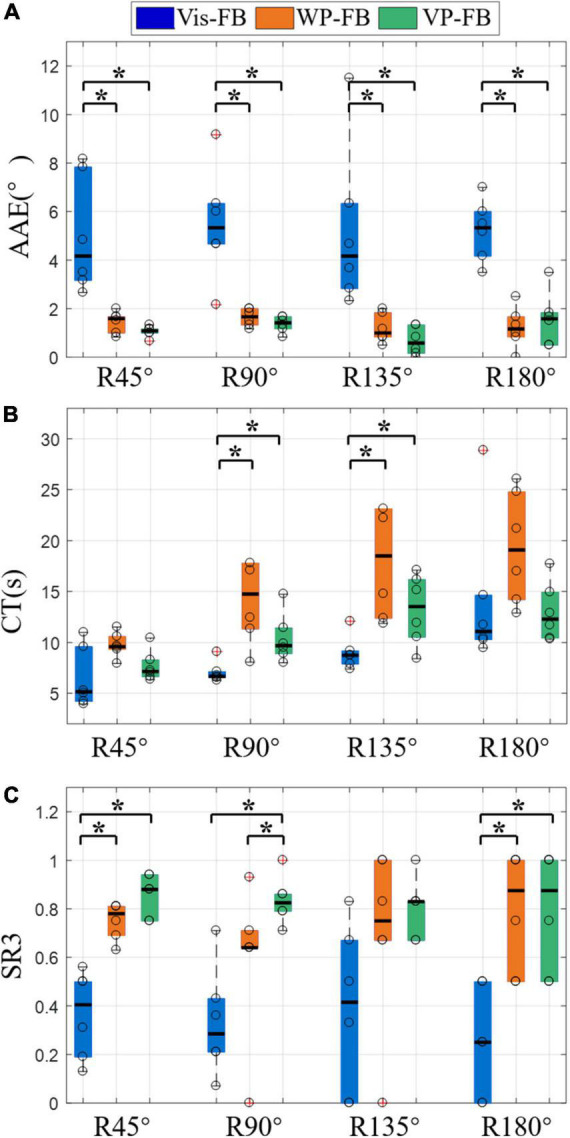
The results of different rotation angles under three feedback methods, **(A)** AAE, **(B)** CT, **(C)** SR3. Boxplots depict the median (*line*), interquartile range (*box*), maximal/minimal values (*whiskers*), and outliers (*crosses*). The hollow circle denotes the median AAE, CT, and SR3 of each subject in panels **(A–C)**, respectively. A star denotes the statistically significant differences (*p < 0.05). AAE, absolute angular error; CT, completion time; SR3, success rate when considering the time and angle thresholds.

The control performance from the third perspective was displayed in Figure 9, which showed the AAE, CT, and SR3 at different rotation angles under three feedback methods. Figure 9A showed that there were significant differences in AAE across three feedback methods for all rotation angles (DoF = 2, χ^2^ = 9.33, 9.33, 9.48, and 10.17 for four rotation angles, respectively, *p* < 0.01). The AAE of Vis-FB was significantly larger than that of WP-FB and VP-FB at all rotation angles, while there was no significant difference between WP-FB and VP-FB. This was in accordance with the performance of AAE of the total movements. However, there was no significant difference among different rotation angles in the same feedback method.

Figure 9B showed that there were significant differences in CT across three feedback methods for R90° and R135° (DoF = 2, χ^2^ = 12.00, and 7.00 for two rotation angles, respectively, *p* < 0.05). The CT of WP-FB was larger than that of Vis-FB and VP-FB at all rotation angles. Besides, the CT of Vis-FB was significantly different from that of WP-FB and VP-FB at R90° and R135°. When comparing the difference in CT in the same feedback method, there were significant differences in CT across four different rotation angles for Vis-FB (DoF = 3, χ^2^ = 33.18, *p* < 0.001), WP-FB (DoF = 3, χ^2^ = 8.58, *p* < 0.05), and VP-FB (DoF = 3, χ^2^ = 12.9, *p* < 0.01). Under Vis-FB, the CT increases with the rising rotation angle. Under WP-FB, the CT at R180° [17.36 (10.59)] was not larger than that at R135° [17.36 (10.76)]. Similarly, under VP-FB, the CT at R180° [12.17 (4.51)] was not larger than that at R135° [12.85 (5.63)]. The CT of VP-FB at R45° and R90° showed a significant difference from that at R135° and R180°.

As shown in Figure 9C, the SR3 of three feedback methods showed significant differences at R45° (DoF = 2, χ^2^ = 10.33, *p* < 0.01), R90° (DoF = 2, χ^2^ = 8.33, *p* < 0.05), and R180° (DoF = 2, χ^2^ = 7.24, *p* < 0.05). The SR3 of WP-FB showed significant differences from that of VP-FB at only R90°. The SR3 of VP-FB was two times larger than that of Vis-FB, and this difference was statistically significant. There is no significant difference in SR3 among different rotation angles in the same feedback method.

## Discussion

### Vibrotactile perception range test

The vibrotactile perception range test showed that the perception range affects the control performance of the arm prosthesis, as displayed in Figure 6. The results showed that the CT decreased with the increase in perception range, which can be mainly attributed to two reasons. One reason is that when the perception range increases, the rotation angle needed to reach the target positions decreases. The other reason is that a larger perception range makes locating the prosthesis easier, which reduces the position adjustment time during the prosthetic control. Unlike CT, the AAE increased with the increase of the perception range. Therefore, a smaller perception range may make the prosthetic control more accurate. However, a smaller perception range may also lead to a higher CT because it asks for more time for subjects to adjust the angular position of the arm prosthesis to move into the preset perception range. Large CT and AAE can both degrade the effectiveness of prosthetic control. In view of the control effectiveness of the prosthesis, this manuscript proposed two parameters, the angular error threshold and the time threshold, to calculate the SR3 of the prosthetic control. There was no significant difference between range 1 and range 2 in AAE, CT, and SR3. Therefore, range 2 was utilized in the WP-FB for its slight advantage in SR3.

The current results were achieved under preset time and angular error thresholds. Various thresholds may lead to different results. Therefore, the thresholds should be decided by the user requirements and design objectives, and the perception range utilized in WP-FB may be changed accordingly. Besides, the value of the minimum perception range analyzed in the test is the subjective choice of subjects. Since the amputees may not have the same skin perception function as normal people, the perception range utilized in the vibrotactile perception range test may be different when the prosthesis is applied to the amputees, which will be studied in the future.

### Sensory feedback test

The effectiveness of the position feedback on prosthetic control was explored in the sensory feedback test. Figure 7 shows that the AAE of Vis-FB was about three times larger than that of WP-FB and VP-FB, which explains the difficulty of making one-time success when visual feedback is the only feedback method used in the prosthetic control. The smallest SR2 of Vis-FB also proves this. The AAE is dramatically reduced when WP-FB is added to the prosthetic control. And this trend happened in all subjects. The AAE of WP-FB is similar to that of VP-FB, demonstrating that adding Vis-FB to WP-FB will not further reduce AAE. Therefore, the WP-FB is the main reason that reduces the AAE during prosthetic control. However, this does not mean Vis-FB is insignificant. On the contrary, Vis-FB is the most natural feedback method and essential in prosthetic control. The CT of the three feedback methods was significantly different pairwise, as shown in Figure 7B. The CT of WP-FB is significantly larger than that of Vis-FB. Due to the lack of intuitive feedback, it takes more time for WP-FB to adjust the arm prosthesis to a target position. The time cost to move the prosthesis within the preset range around target angular positions makes the WP-FB has the largest CT and smallest SR1. The CT of VP-FB was significantly reduced compared to that of WP-FB by adding Vis-FB to WP-FB. However, the CT of VP-FB was still significantly larger than that of Vis-FB in some subjects. This may be because when Vis-FB and WP-FB were both utilized in the prosthetic control, the subject attempted to use Vis-FB to make the prosthesis close to the target positions before accurately moving it within the preset range with WP-FB. The process of adjusting the angular position of the prosthesis increased the CT during prosthetic control. The SR3 of the three feedback methods were significantly different pairwise, as shown in Figure 7C. Adding WP-FB to Vis-FB can dramatically improve the SR3 while affecting little on SR1. This demonstrates that WP-FB is necessary for accurate prosthetic control, especially for tasks with strict angular position requirements, such as opening the door with a key.

Figure 8 showed that when movements are grouped according to the target positions, the AAE of different positions were in accordance with that when considering all movements together, indicating that the influence of feedback methods on the AAE is not affected by target positions. Only the AAE is compared because the rotation angles required to reach target positions vary, affecting the CT of each position.

Figure 9A showed that when movements are grouped according to the rotation angles, the AAE at different rotation angles were in accordance with that when considering all movements together, which demonstrates that the influence of feedback methods on the AAE is not affected by rotation angles. The CT under three feedback methods was distinct at different rotation angles, as shown in Figure 9B. It can be seen that the CT of Vis-FB increases with the rising rotation angle. In contrast, when it comes to the WP-FB and VP-FB, the increasing rotation angle does not necessarily lead to the rise of CT. For example, the CT of R180° is not larger than that of R135° under both WP-FB and VP-FB. This may be due to the presence of WP-FB, which affects the CT by adding the adjustment process to the prosthetic control. Figure 9C showed that the SR3 of WP-FB showed significant differences from that of VP-FB at R90°, indicating that Vis-FB helps locate the prosthesis at some time. The SR3 of Vis-FB is about half less than that of VP at most rotation angles, demonstrating that accurate prosthetic control is hard to complete with only Vis-FB. Besides, no significant difference existed in SR3 among different rotation angles in the same feedback method, proving that SR3 of all three feedback methods is not affected by rotation angles.

As a whole, WP-FB helps improve the control performance of the myoelectric prosthesis by significantly reducing the angular error and improving the success rate of prosthetic control. However, the time and angular error thresholds set up in this manuscript may affect the results of the prosthetic control, which would be decided by the user requirements and design objectives when used practically by amputees and studied in the future.

## Conclusion

This manuscript designed a myoelectric arm prosthesis with WP-FB. To study the effect level of position feedback on prosthetic control, two tests were performed. The vibrotactile perception range test aims to analyze the perception sensitivity of the vibration in humans and obtain the optimal perception range utilized in the WP-FB. The sensory feedback test analyzes the effectiveness of the position feedback by comparing three feedback methods of Vis-FB, WP-FB, and VP-FB. Our experimental results showed that adding WP-FB to Vis-FB can significantly reduce angular error and improve the success rate of prosthetic control. When WP-FB was added to the prosthetic control, the AAE of the prosthetic wrist declined from 4.50° to 1.08°, while the SR3 increased from 0.34 to 0.84. This study demonstrates the importance of WP-FB to the effective control of the arm prosthesis. However, this manuscript did not study the effect mechanism of position feedback and the influence of the thresholds on the parameter selection, which will be explored in a future study.

## Data availability statement

The raw data supporting the conclusions of this article will be made available by the authors, without undue reservation.

## Ethics statement

The studies involving human participants were reviewed and approved by the Institutional Review Board of Shenzhen Institute of Advanced Technology, Chinese Academy of Sciences. The patients/participants provided their written informed consent to participate in this study. Written informed consent was obtained from the individual(s) for the publication of any potentially identifiable images or data included in this article.

## Author contributions

YZ, LT, and XL contributed to the conception and design of the study. YT and ZY made part of the devices. YZ performed the statistical analysis and wrote the first draft of the manuscript. All authors contributed to manuscript revision, read, and approved the submitted version.

## Conflict of interest

The authors declare that the research was conducted in the absence of any commercial or financial relationships that could be construed as a potential conflict of interest.

## Publisher’s note

All claims expressed in this article are solely those of the authors and do not necessarily represent those of their affiliated organizations, or those of the publisher, the editors and the reviewers. Any product that may be evaluated in this article, or claim that may be made by its manufacturer, is not guaranteed or endorsed by the publisher.
